# Supplementary figures and images for: Dynamics of Trypanosoma cruzi infection in hamsters and novel association with progressive motor dysfunction
Source: PLoS Negl Trop Dis. 2024 Jun 21;18(6):e0012278. doi: 10.1371/journal.pntd.0012278 (PMC11221660; doi:10.1371/journal.pntd.0012278)

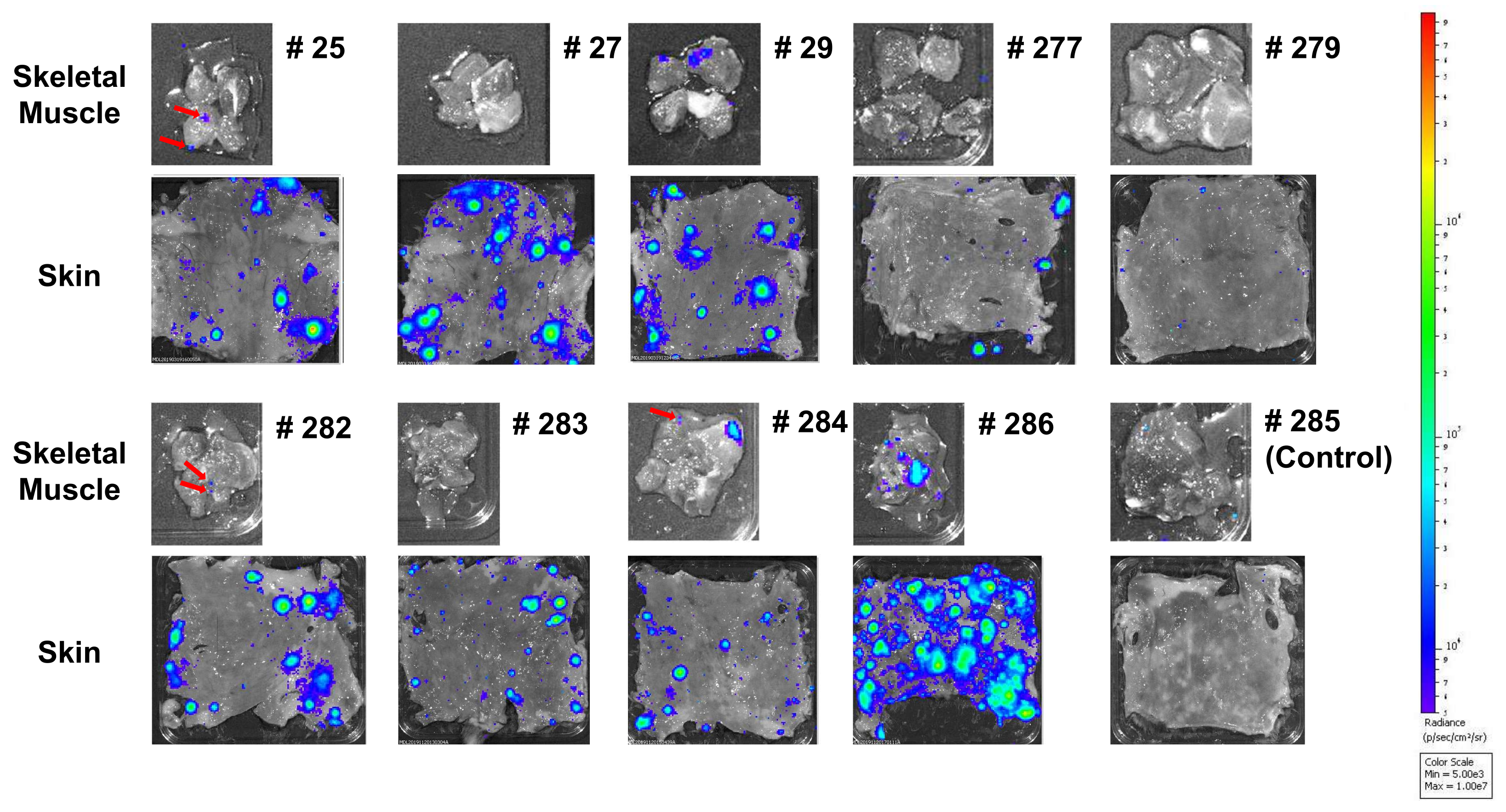

Supplement: S1 Fig — Images for each hamster were taken immediately post-mortem at at 152–182 days post-infection. Numbers are individual animal codes. Images from one representative not infected control are shown for comparison. Log-scale pseudocolour heat-map shows intensity of bioluminescence as a proxy for parasite load; minimum and maximum radiances are indicated. Red arrows highlight small foci of bioluminescence. (TIF) [file pntd.0012278.s001.tif]

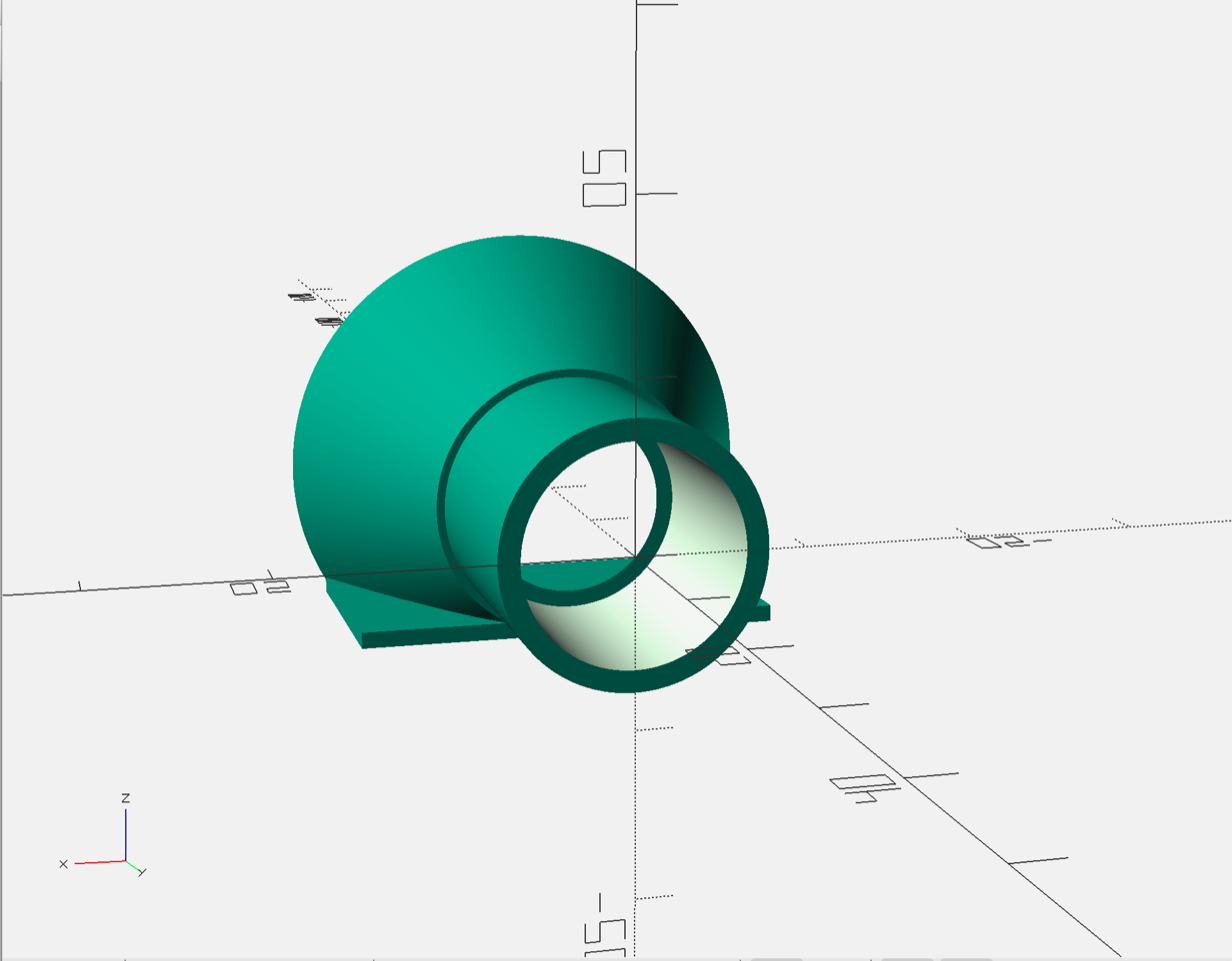

Supplement: S1 Data — (ZIP) [file pntd.0012278.s002.zip › Nose_cone_back.png]

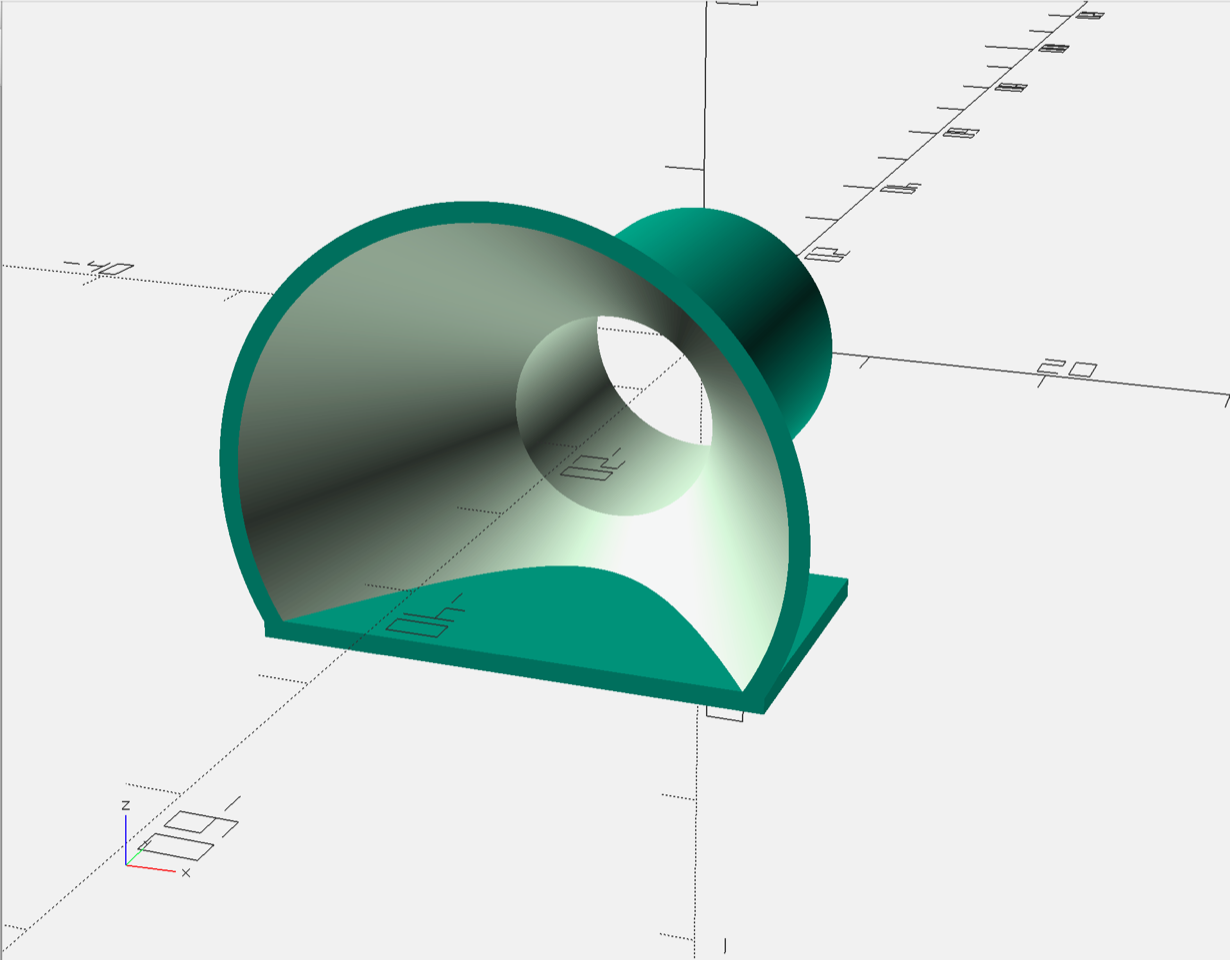

Supplement: S1 Data — (ZIP) [file pntd.0012278.s002.zip › Nose_cone_front.png]
