# Supplementary material for: Dynamics of Trypanosoma cruzi infection in hamsters and novel association with progressive motor dysfunction
Source: PLoS Negl Trop Dis. 2024 Jun 21;18(6):e0012278. doi: 10.1371/journal.pntd.0012278 (PMC11221660; doi:10.1371/journal.pntd.0012278)
Supplement: Attachment — Submitted filename: Hamster ms_response to reviewers comments.pdf [file pntd.0012278.s004.pdf]

PNTD-D-23-01430 (Langston et al) – Authors' responses to reviewers' comments

We have reproduced below the verbatim reviewers' reports and highlighted in yellow the points requiring follow up. Our responses are in red text and the line numbers given are for the 'clean' version of the revised manuscript.

The Reviewers and Editors acknowledge the relevance of your report showing the hamster as an alternative experimental model for Chagas disease. Even though cardiac and digestive track pathologies associated with human Chagas disease are not well reproduced in the hamster model, this new model offers new opportunities for studies of peripheral nervous system involvement in Chagas disease. This report is well within the scope of PNTD. However, please address all the comments and questions of the reviewers in your revised manuscript.

Reviewer's Responses to Questions

Key Review Criteria Required for Acceptance?
As you describe the new analyses required for acceptance, please consider the following:

Methods
-Are the objectives of the study clearly articulated with a clear testable hypothesis stated?
-Is the study design appropriate to address the stated objectives?
-Is the population clearly described and appropriate for the hypothesis being tested?
-Is the sample size sufficient to ensure adequate power to address the hypothesis being tested?
-Were correct statistical analysis used to support conclusions?
-Are there concerns about ethical or regulatory requirements being met?

Reviewer #1: -Are the objectives of the study clearly articulated with a clear testable hypothesis stated?
YES
-Is the study design appropriate to address the stated objectives?
YES
-Is the population clearly described and appropriate for the hypothesis being tested?
YES
-Is the sample size sufficient to ensure adequate power to address the hypothesis being tested?
YES
-Were correct statistical analysis used to support conclusions?
YES
-Are there concerns about ethical or regulatory requirements being met?
NO

Reviewer #2: -Are the objectives of the study clearly articulated with a clear testable hypothesis stated? yes
-Is the study design appropriate to address the stated objectives? yes
-Is the population clearly described and appropriate for the hypothesis being tested? yes
-Is the sample size sufficient to ensure adequate power to address the hypothesis being tested? yes
-Were correct statistical analysis used to support conclusions? yes
-Are there concerns about ethical or regulatory requirements being met? No

For the fibrosis index information is missing as to how many fields were analysed for each image. For both the inflammatory and fibrosis indices it is not clear if all fields of view were in the same tissue slice or if multiple tissue slices were analysed per animal. Please add this information in the methods (lines 142 – 148).

The approach was the same as for the inflammation analysis, using 10-15 fields of view at 400X magnification. The methods section has been edited to make this clear (section starting at line 139).

Line 144: “inflammation index” – I assume this is the same as the myositis score, please clarify/make consistent in the methods section.

Yes, we now use myositis (for skeletal muscle) and myocarditis (for heart) score in the methods to match the terminology used in the figures.

Results
-Does the analysis presented match the analysis plan?
-Are the results clearly and completely presented?
-Are the figures (Tables, Images) of sufficient quality for clarity?

Reviewer #1: -Does the analysis presented match the analysis plan?
YES
-Are the results clearly and completely presented?
YES
-Are the figures (Tables, Images) of sufficient quality for clarity?
YES

Reviewer #2: -Does the analysis presented match the analysis plan? yes
-Are the results clearly and completely presented?

Line 206: limb adduction: I think it would benefit the reader to give a brief explanation here

of what limb adduction is.

Phrase added (line 215-6) to clarify this means movement towards the body's midline.

Line 277: hindlimb parasite bioluminescence in half of the animals at day 152-182. This data does not appear to be presented in the results section. Considering the importance of this data with respect to the adduction phenotype, hindlimb skeletal muscle associated bioluminescence should be shown in Figure 2 B to G, or as a separate panel in figure 4.

The data are in figure 2G, but we do see that by showing them alongside the data for so many other tissue types that they are a bit lost. To give the skeletal muscle parasite loads more prominence we now include all of the *ex vivo* bioluminescence images for skeletal muscle in a supplementary figure (S1 Fig) alongside all the skin images for comparison. We also edited the text to be more precise: "approximately half of animals" changed to "five of nine (55.6%) animals" (Line 290).

-Are the figures (Tables, Images) of sufficient quality for clarity? Yes

----

Conclusions
-Are the conclusions supported by the data presented?
-Are the limitations of analysis clearly described?
-Do the authors discuss how these data can be helpful to advance our understanding of the topic under study?
-Is public health relevance addressed?

Reviewer #1: -Are the conclusions supported by the data presented?
YES
-Are the limitations of analysis clearly described?
YES
-Do the authors discuss how these data can be helpful to advance our understanding of the topic under study?
PARTIALLY
-Is public health relevance addressed?
PARTIALLY

Reviewer #2: -Are the conclusions supported by the data presented? Yes, except: the authors show inflammation and fibrosis in the quadriceps of infected hamsters. To determine if inflammation and fibrosis are directly linked to the gait abnormalities in infected hamsters the authors checked mouse muscle tissues for inflammation and fibrosis, and found clear markers of inflammation and fibrosis in mouse myocardium. As mice do not show gait

abnormalities, that authors conclude that the gait abnormalities are likely not the direct result of parasite induced inflammation and fibrosis. However, different tissues are being assessed in mice compared to hamsters. The relevant tissue for the gait abnormality is the skeletal muscle of the hindlimb (e.g. quadriceps). For the argument to be robust the authors should investigate the levels of inflammation and fibrosis in mouse quadriceps, as it is possible that infected mice do not show inflammation and fibrosis in their hindlimbs. I suggest that either histopathological data for mice quadriceps are included, or a caveat is included in the discussion.

We were comparing like with like i.e. skeletal muscle tissue samples from both mice and hamsters were analysed. The reviewer's concern arises from a mistake that we made in the legend for figure 6, parts of which had been copy-pasted from an older manuscript in which heart tissue from the same mice had been analysed. We failed to edit this legend in several places, which has now been done to make clear the analysis was of hindlimb skeletal muscle (legend now at line 577). Apologies for this oversight.

-Are the limitations of analysis clearly described? A limitation of the study is that only one strain was assessed. The value of carrying additional studies with different strains should be covered briefly in the discussion.
-Do the authors discuss how these data can be helpful to advance our understanding of the topic under study? yes
-Is public health relevance addressed? Yes

Editorial and Data Presentation Modifications?
Use this section for editorial suggestions as well as relatively minor modifications of existing data that would enhance clarity. If the only modifications needed are minor and/or editorial, you may wish to recommend "Minor Revision" or "Accept".

Reviewer #1: (No Response)

Reviewer #2: According to the PLOS data availability policy the following data should be provided:

- The values behind the means, standard deviations and other measures reported;
- The values used to build graphs;

This data is not provided for the manuscript.

These source data are now provided in a supplementary file: S2 Data.xlsx

----

Summary and General Comments
Use this section to provide overall comments, discuss strengths/weaknesses of the study, novelty, significance, general execution and scholarship. You may also include additional comments for the author, including concerns about dual publication, research ethics, or publication ethics. If requesting major revision, please articulate the new experiments that are needed.

Reviewer #1: SUMMARY

The aim of this study was to develop an in vivo/ex vivo bioluminescence imaging system for Trypanosoma cruzi (T. cruzi) infection in a hamster model.

The mouse animal model has been widely used in Chagas disease (CD) research. Chronically T. cruzi infected mice develop cardiac and gastrointestinal pathologies reproducing some clinical features of the disease in humans.

A better understanding of Chagas disease pathogenesis was possible with the development of highly sensitive bioluminescence imaging based on the use of T. cruzi parasites that express a red-shifted luciferase. These investigations allowed the study of parasite dissemination and infection dynamics in mice.

In this paper, the authors applied the bioluminescence imaging technology to study *T. cruzi* infection in the hamster model, which shows a close alignment with human diseases for several pathogens such as *Leishmania donovani* among others.

In contrast to what has been found in the mouse model, chronically infected hamsters did not show signs of heart or gut disease. The infection was restricted to the skin and subcutaneous adipose tissue. Interestingly, they exhibited stiffness in the hindlimbs that resulted in an altered gait, suggestive of nervous system damage.

MERITS

Few research groups have studied CD in the hamster model. This is the first investigation that monitors T. cruzi infection over time and clinical manifestations in large rodents, using bioluminescence imaging. Although preliminary, this work provides evidence of peripheral nervous system damage in chronic CD and may be valuable for future research on this unique and not well studied pathological outcome of the disease. However, the translational value of this model needs to be further explored, mainly because humans do not develop similar locomotive dysfunctions.

COMMENTS

1. Introduction:

a) Line 64: please include a few references about the models suitable for studying aspects of Chagas digestive disease.

We have added four references to this section (line 64).

b) Line 69: as the authors mentioned, one manifestation of CD is the presence of parasites in the central nervous system in the acute phase or due to reactivation during chronic disease. Indeed, patients with cerebral CD may have symptoms such as confusion, headaches, convulsions and altered mental status (recently reviewed by Shelton WJ, Gonzalez JM. "Outcomes of patients in Chagas disease of the central nervous system: a systematic review". Parasitology. 2023 Nov 21:1-9. doi: 10.1017/S0031182023001117). I suggest the authors to expand this paragraph and add information on this important clinical aspect involving the nervous system in humans.

We have cited the very useful and relevant article recommended by the reviewer. We decided to retain the focus on animal models in this particular section, but we have added further text in the discussion on clinical presentation in human infections with CNS involvement where we felt this was a better fit (lines 303-306).

2. Methods:

c) Line 109: please indicate the source of the Living Image v4.7 software.

Added PerkinElmer as the source.

d) Lines 121-122: please provide a link or reference for the detection threshold method.

We have cited the original paper on the development of the imaging model in mice (Lewis et al., 2014).

e) Line 123: please indicate which version of the Living Image software you used.

The version number has been added.

f) Line 144: please provide a link or reference for the inflammation index calculation.

g) Line 146: please provide a link or reference for the fibrosis index calculation.

We added citations for Caliari (1997) and Caliari et al (2002).

h) Please add a sub-section to describe how you evaluated the limb abduction with

references.

This was done using the photos from the bioluminescence imaging sessions. We have added a new section "Limb adduction evaluation" to the methods to describe this (line 155). The approach was devised by the authors and simply involved measuring the distance between fore/hind-paws, so we have not included a reference.

3. Results:

i) Figure 3: please indicate/circle the inflammation and fibrosis regions in the infected heart tissue.

We have added some annotations as suggested and updated the figure legend accordingly.

j) Line 208: the authors state that "the phenotype was highly significant and developed in 100% of chronically infected hamsters". The results shown in Figure 4B do not reflect that statement. Please explain.

Apologies, this should have referred to Figure 4D, not 4B. This has been corrected (line 218-9).

k) Line 223: please clarify and expand the sentence "A neuropathy aetiology therefore seems probable".

We decided to remove this sentence from the results because we felt that explaining this conclusion would be more appropriate in the expanded discussion (line 293 onwards) prompted by the reviewer's other comments (specifically, b), o) and p)).

l) Figure 5: same as in i).

We have added some annotations as suggested and updated the figure legend accordingly.

m) Figure 6: please add the bars corresponding to the myositis score and % collagen obtained in the hamster study to facilitate the comparison.

We have added the hamster data to these figure panels, but note that the baselines are different for mice and hamsters, which is why we focused on fold changes in the results text.

4. Discussion:

n) Line 269: the authors acknowledge the evolutionary advantage of skin-resident parasites populations and the impact on T. cruzi transmission. They have found that the skin is a predominant parasite reservoir in chronically infected hamsters. This finding is important

in the context of CD transmission and highlights the utility of the hamster model. I encourage the authors to expand this paragraph to mention this remarkable observation.

We are pleased that the reviewer shares our enthusiasm about this part of the study. We already devoted 14 lines of discussion to the skin data, so we have limited ourselves to adding a sentence highlighting some important questions that need to be addressed about skin parasite developmental form(s) and cell tropism (line 281-284).

o) Line 290: what is spastic displegia? why will research on spastic displegia be relevant? Please explain and cite a reference.

Our idea was that our model might have some relevance to the cerebral palsy field e.g. it may have some value for investigating fundamental mechanisms of tissue damage that result in spasticity as a functional outcome. We have extended the sentence and added a reference to support our line of thinking; it now reads "It may also prove relevant to wider research on spastic diplegia (bilateral lower limb muscle tightness), a common presentation of cerebral palsy that lacks a predictive small animal model (Clowry et al)." (Line 310-312)

p) Please expand the last paragraph to further explain why you think this model could be useful to explore peripheral nervous system damage in chronic CD although humans do not develop similar symptoms.

Our analysis of pathology was restricted to inflammation, fibrosis and quantifying limb adduction, but these may just be the most obvious manifestations in the hamster – there may be other, more subtle impacts on the PNS that we have not tested for but with a closer match to human symptoms. We extended the sentence "Skeletal muscle denervation, neuro-muscular junction, microvascular, mitochondrial and metabolic abnormalities have all been described" with the phrase "so it would be interesting to analyse whether these occur in *T. cruzi*-infected hamsters" (Line 201).

Reviewer #2: In this manuscript the authors develop a bioluminescent hamster model to assess its value for Chagas disease research, in particular with respect to parasite distribution and pathological outcomes. The study shows that longitudinal imaging of the infection is possible in hamsters, although detection limit may be worse than in mice due to larger size of the animals and organs. In the acute phase parasites are widely distributed as seen in other models. Interestingly in the chronic phase parasites reside mainly in skin, and also other fatty tissues, and the GI tract does not appear to be a reservoir unlike in mice.
In line with the distribution pattern, a low rate of cardiomyopathy was detected, lower than seen in mice. While this may align with the human situation where cardiomyopathy is only detected in a subset of people, a question is how much this a Tc CLBR strain specific effect. As mentioned by the authors previous studies with hamsters have shown more frequent cardiomyopathy.

Overall, the presented model is not deemed suitable for the study of cardiac and digestive Chagas pathology. However, the study did identify a previously not observed Chagas disease pathology affecting the hindlimbs and gait. While this is not a symptom of human disease there may be value in understanding the underlying pathology and its translational value. This work is of interest to the general Chagas disease community, including those interested in models of human pathophysiology and drug discovery.
